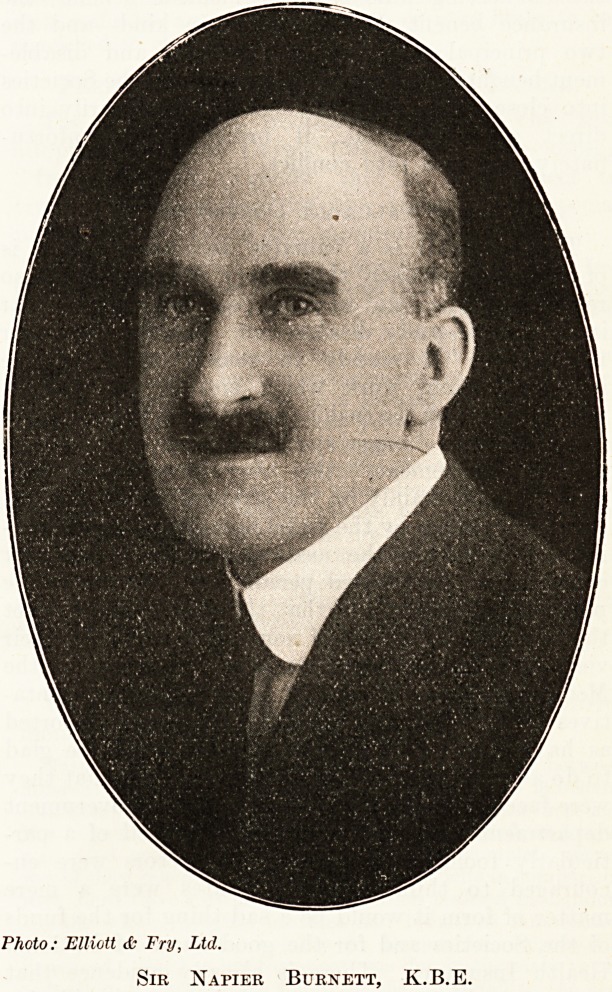# The Hospital and Nursing Exhibition

**Published:** 1923-04

**Authors:** 


### THE HOSPITAL EXHIBITION.

### AN ATTRACTIVE PROGRAMME.

SOME distinguished lecturers have been secured for the conferences at the Hospital, Nursing and Midwifery Exhibition which opens on April 3 at the Central Hall, Westminster, and at each lecture the chair will be taken by a matron of one of the great London hospitals. At 3 p.m. on the opening day Mr. Godfrey H. Hamilton, Secretary of the National Hospital for the Paralysed and Epileptic, will lecture on "Hospital Administration," with cinematograph illustrations. At 8 p.m. Colonel Nathan Raw will speak on "The Relation of Physical Disease to Insanity," and on the following afternoon at 2.30 p.m. the lecturer will be Mr. Aleck Bourne, the well-known obstetrician. At 8 p.m. Mr. E. B. Turner, F.R.C.S., Vice-President of the National Council for Combating Venereal Disease, will speak on "Venereal Disease, Its Medical, Social and Prophylactic Aspects," and the Medical Officer of Health for Edmonton, Dr. Harding, will take as his subject "The Rôle of the Midwife in the Public Health Scheme." Other lectures include "Serums, Vaccines and Allied Products," by Mr. Stanley White, M.R.C.S., L.R.C.P., at 2.30 p.m. on April 5; "Infant Feeding," by Dr. Robert Hutchison, of the London Hospital, at 8 p.m. on the same day; and "Tuberculosis and Its Relation to National Life," by Mr. R. C. Wingfield, Medical Superintendent of Brompton Hospital Sanatorium, on April 6 at 2.30. At 8 p.m. on that evening Dr. Saleeby will speak on "Sunlight and Health."

There will be a number of interesting exhibits—a model operating theatre, a model matron's office where filing systems will be displayed, and a model ward-room and kitchen equipment. The British Red Cross are showing the equipment in use at their London clinics for orthopædic treatment, including local light baths, clinic switch table, and vibratory massage apparatus. There will be an exhibit showing some of the activities of the St. John's Ambulance Association and of the Administration de l'Assistance Publique of Paris. The reception on the opening day will begin at 2.30 and the Reception Committee includes Sir Arthur Stanley, G.B.E., Sir Napier Burnett, K.B.E., the Dowager Lady Balfour of Burleigh and the Hon. Lady Acland. Tickets for the Exhibition may be obtained from the Secretary at 22 and 24 Great Portland Street, W.1. Notices of the exhibits will be found elsewhere.

*Photo: Elliott & Fry, Ltd.*

THE HON. SIR ARTHUR STANLEY, G.B.E.

*Photo: Elliott & Fry, Ltd.*

SIR NAPIER BURNETT, K.B.E.

## Figures and Tables

**Figure f1:**
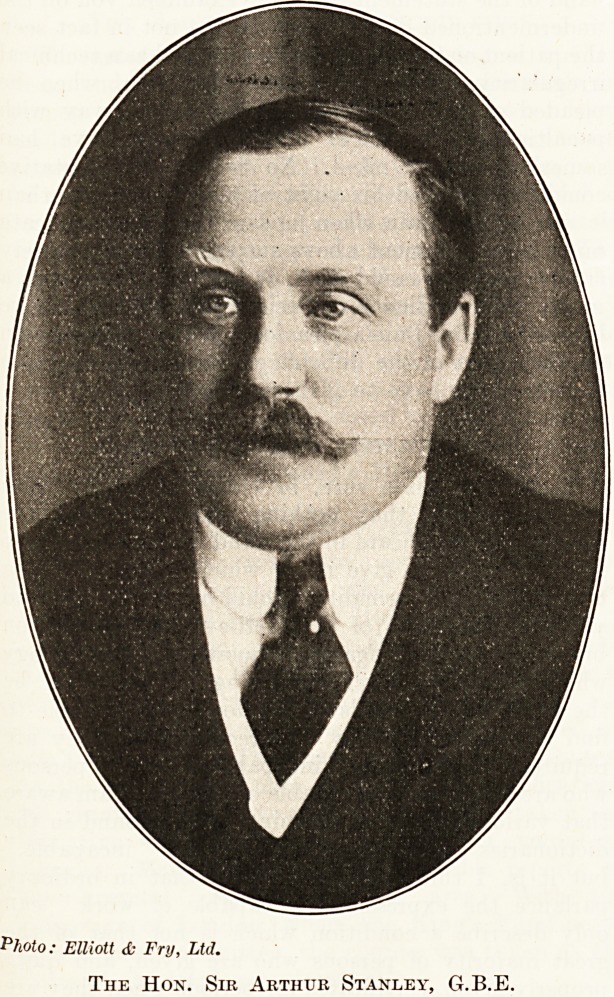


**Figure f2:**